# Application of a tool for the evaluation of public and patient involvement in research

**DOI:** 10.1136/bmjopen-2014-006390

**Published:** 2015-03-13

**Authors:** Susan Jill Stocks, Sally J Giles, Sudeh Cheraghi-Sohi, Stephen M Campbell

**Affiliations:** NIHR Greater Manchester Primary Care Patient Safety Translational Research Centre, Centre for Primary Care, Institute of Population Health, University of Manchester, Manchester, UK

**Keywords:** PRIMARY CARE, STATISTICS & RESEARCH METHODS

## Abstract

**Objectives:**

Public and patient involvement (PPI) is required at all stages of research by many funding bodies such as the National Institute for Health Research (NIHR). Given the high priority of PPI within NIHR programmes and the associated costs, it is important that the process of involvement and impact of PPI on health services research is evaluated. We aimed to develop a tool to quantitatively evaluate the quality of PPI in research from a PPI participant's perspective in order to inform the researchers about absolute level of quality (cross-sectional aspect) and changes in quality over time (longitudinal aspect).

**Setting:**

A primary care patient safety translational research centre.

**Participants:**

The 12 members of the Research User Group (RUG) of Greater Manchester Primary Care Patient Safety Translational Research Centre.

**Interventions:**

By their own choice each RUG member supported a specific research theme. The level of involvement varied from commenting on documents through to designing their own research projects.

**Primary and secondary outcome measures planned:**

Measure absolute score and change in score over time in a nine-point Likert score within individuals. Compare Likert scores before undertaking PPI with scores after PPI activities. Evaluate the usefulness of a questionnaire based on a theoretical framework of personal and research factors.

**Results:**

The questionnaire had an acceptable to good level of internal consistency (Cronbach's α 0.74–0.81). The majority of the individuals met their initial expectations (11/12) and scored high across all factors. There was no significant change over time in the aggregate score over all factors and all individuals, but there were differences within individuals and factors. A ceiling effect limited the questionnaire's usefulness to measure increasing scores.

**Conclusions:**

The questionnaire has been useful in evaluating the early stages of a PPI group and may be generalisable to another setting.

**Strengths and limitations of this study**

- We have used questions based on a framework for quality in public and patient involvement (PPI) to quantitatively evaluate PPI in research for the first time.
- The questionnaire showed good internal consistency between factors identified in a theoretical framework and was discriminatory in identifying individuals with decreasing scores for the quality of their experience of PPI.
- Using a within-subject random effects regression analysis allows an estimate of overall change in score allowing monitoring of overall PPI quality even though individual PPI participants may enter or leave the PPI group.
- A ceiling effect made the questionnaire less useful for measuring increasing scores.
- Evaluating the Cronbach's α in another PPI group is needed to increase the confidence in the internal consistency of the questionnaire.

## Background

The National Institute for Health Research (NIHR) programmes require active involvement of patients and the public (public and patient involvement, PPI) at all stages of research, for example, in the choice of research topics, assisting in the design, advising on the research project or in carrying out the research.^1^ In the same way that the public have a right to have a say about services that are provided for them, they also have an ethical right to oversee and influence the direction taken by research into healthcare provision.^2^ ^3^ The Greater Manchester Primary Care Patient Safety Translational Research Centre (Greater Manchester PSTRC) is an NIHR-funded Research Centre addressing patient safety in primary care settings.^4^ Within the Greater Manchester PSTRC, after an open public call for recruitment, a Research User Group (RUG) was established in September 2013 to fulfill both a strategic governance role and contribute PPI to research activities. Given the high priority of PPI within Greater Manchester PSTRC and NIHR programmes, it is important that the process of engagement and impact of PPI on health services research is evaluated. Although researchers, members of the public and policymakers believe that it is possible to evaluate the impact of PPI on research, it rarely happens.^5^

An individual has many personal reasons for being involved in PPI and these can be encouraged or discouraged by the structures or processes of research and/or the relationships with the researchers. A published theoretical framework aiming to assess the quality of PPI in a research context identified the underlying concepts or factors outlined in box 1.^6^ We aim to use the questions provided with this framework to quantitatively evaluate the quality of the PPI within the RUG that may be generalised to other settings. This research aims to address the broader questions below.

1. Do the questions address the same underlying concept as defined by the theoretical framework?
2. Have the *a priori* expectations of the PPI participants been met?
3. How well is PPI functioning in terms of personal and research factors?
4. Is the quality of the PPI changing over time?
5. How strongly are the scores for personal factors associated with those for research factors?

## Methods

The RUG consists of 12 members with an elected chair that met every 4–6 weeks between September 2013 and April 2014 (6 times in total). RUG members each support a specific research theme (by their own choice from medication safety, multimorbidity, general practice, interface and informatics) or the core theme which focuses on administration and PPI. Expenses are paid at the INVOLVE rate which depends on the individual circumstances and the nature of the task (eg, the daily committee fee is £150).^7^ RUG members may be involved at all levels and stages of research from commenting on documents to designing their own projects.

Box 1 **Quality involvement framework factors in evaluating the quality of public and patient involvement (PPI) in research^6^**

Personal factors

1. Being valued, for example, being paid and treated hospitably
2. Achieving one's own goals through involvement
3. Feeling able to make a contribution (empowered)

Research factors (relationships and ability to participate)

- A clear role for PPI in research and supportive structures, for example, motivated researchers, adequate funding and access to guidance on the processes of research
- Support at the organisational level and by existing ethical and governance systems
- Application of previous experience as a service user or supporting research

The questionnaire^6^ was adapted to the specific context of the Greater Manchester PSTRC and a further question assessed whether or not the PPI group (RUG) followed the ground rules that they developed among themselves (see online supplementary appendix 1). Whether or not the RUG members met their own expectations was assessed by comparing an expectations questionnaire with the evaluation questionnaire. The expectations questionnaire consisted of 12 questions adapted from the evaluation questionnaire by replacing “Are you able to…” with “Do you expect to be able to…”, etc (denoted E in online supplementary appendix 1). The expectations questionnaire was completed before the first RUG meeting and the evaluation questionnaire was completed online within 1 week of each RUG meeting. The question order was randomised for each individual and each administration.

Responses were measured on a nine-point Likert scale. In order to address research question 1, the internal consistency of the responses within each factor on the first administration was estimated by Cronbach's α. To address research question 2, a paired t test was used to compare the score in the expectations questionnaire with the mean score across all the surveys for each question within each individual. Research question 3 was addressed by reporting the mean response scores over all six surveys at the level of each factor (1–5, box 1) and each participant, each factor across all participants and across all factors and all participants. Research question 4 was addressed by estimating the change in response score using multilevel mixed effects linear regression models with survey number as the predictor in Stata V.13. For estimates of change within individuals and factors, a two-level model where the dependent variable was the response score nested within questions (the random effects or higher level in the model) was used. For estimates of change across all individuals, a similar three-level model included random effects on question numbers and individuals, that is, the response variable was nested within questions nested within the individuals. The results are presented as the change in response score relative to the first survey (assuming a linear trend) over the six surveys for each factor and across all factors. The question about adherence to the RUG ground rules (Q22, see online supplementary appendix 1) was a single item Likert-type scale; therefore, a non-parametric approach was taken (Kendall τ rank correlation coefficient).^8^ To address research question 5, the mean response scores for personal factors within surveys and individuals were compared with the scores for research factors using a three-level mixed effects regression model. The dependant variable was the mean response score for personal factors nested within survey number and individuals (random effects) with mean response score for research factors as the predictor.

The use of regression models to analyse Likert scale data remains a long-standing debate.^9^ Arguably these data might be less likely to violate the assumptions of a linear regression in that it is truly Likert scale data as it uses several questions to address the same underlying concept and the wider nine-point scale was used. However, the analysis was repeated using an ordered logistic regression model (ologit in Stata) to check that the assumptions made by the linear regression did not substantially alter the results. The advantage of the linear regression is the capacity to include random effects using Stata, that is, to allow each individual to vary independently. An interim analysis was undertaken after three administrations of the questionnaire and feedback was provided to the researchers and the RUG.

All members of the RUG gave informed consent for the evaluation. This paper was circulated among the RUG and their comments are considered in the discussion.

## Results

The questionnaire performed well across all factors with an acceptable to good level of internal consistency within each factor 1–5 (Cronbach's α 0.74–0.81) for survey 1. Out of 1159 potential responses to questions 1–19, 86 (7%) were answered ‘not applicable’, these were distributed equally across the questions and omitted from the analysis. Just one RUG member expressed difficulty in understanding the meaning of questions 3, 7, 11, 12 and 20.

The expectations questionnaire was completed by 11/12 (92%) RUG members and the survey completed 61 times out of 65 potential completions (94%). RUG members had high *a priori* expectations (mean overall score 7.2, table 1) and these expectations were largely met (mean score over all members and all surveys 7.3, table 1). However, one individual's experience did not meet their initial expectations (8.2, cf. 7.1, p=0.02; individual 5, table 1); this member subsequently resigned.

**Table 1:** Comparison of mean expectations and evaluation survey scores

ID*	Number of surveys	Mean score expectations±SD	Mean score surveys 1–6±SD	p Value Paired t test (by question)
1	5	7.6±0.33	8.0±0.13	0.28
2	6	6.4±0.47	6.1±0.20	0.67
3	6	6.9±0.38	7.4±0.11	0.24
4	6	8.3±0.47	7.7±0.15	0.32
5†	5	8.2±0.41	7.1±0.18	**0**.**02**
6	6	6.8±0.69	7.6±0.17	0.35
7	6	6.1±0.87	7.6±0.13	0.15
8†	2	6.2±0.55	5.1±0.37	0.15
10	6	7.2±0.37	7.0±0.16	0.63
11†	4	8.6±0.31	8.4±0.07	0.48
12	5	6.9±0.60	7.7±0.18	0.16
All	6	7.2±0.17	7.3±0.06	0.97

Bold typeface indicates significance at p<0.05.

*One individual did not complete the expectations questionnaire.

†Members who resigned from the Research User Group during the analysis period.

**Figure 1:**
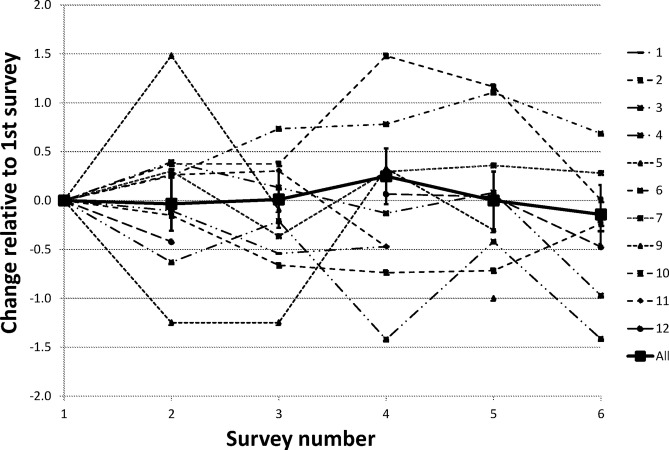
Changes in Likert score over time within individuals and over all individuals.

The whole group score over all factors was high (7.3±0.04, table 2), and over all RUG members and factors, there was no significant change in score (−0.02, −0.06 to 0.02; table 2). The estimated change in individual scores and across the whole group is shown in figure 1. At the individual level, there were three individuals showing an overall decreasing trend and one with an increasing trend (3, 9, 11, 6; table 2).

**Table 2:** Mean scores and change in score over all evaluation surveys (1–6) within individuals and across the group

ID	Number of surveys	Being valued (Q1–6)		Achieving own goals (Q7–9)		Empowered (Q10–12)		Research relationships and level of participation (Q13–19)		Experience as a service user or supporting research (Q20–21)		All factors (Q1–19)		Follow ground rules (Q22)	
		Mean ±SD	Mean change 95% CI	Mean ±SD	Mean change 95% CI	Mean ±SD	Mean change 95% CI	Mean ±SD	Mean change 95% CI	Mean ±SD	Mean change 95% CI	Mean ±SD	Mean change 95% CI	Median range	τ-b p Value
1	5	8.2±0.16	**−0.21** **−0.36 to −0.06**	8.4±0.25	−0.03 −0.27 to 0.22	8.1±0.25	+0.08 −0.20 to 0.37	7.8±0.18	−0.03 −0.20 to 0.13	9.0±0.00	–	8.1±0.10	−0.07 −0.17 to 0.03	7 4–8	0.55 0.47
2	6	5.2±0.29	+0.01 −0.33 to 0.36	6.2±0.36	+0.34 −0.02 to 0.71	6.4±0.47	+0.29 −0.01 to 0.56	6.3±0.25	−0.07 −0.27 to 0.13	8.2±0.47	+0.00 −0.26 to 0.27	6.0±0.16	+0.08 −0.07 to 0.23	7 6–7	–
3	6	7.3±0.17	**−0.31** **−0.48 to −0.15**	7.1±0.22	−0.14 −0.37 to 0.08	7.3±0.24	−0.13 −0.31 to 0.05	7.5±0.14	**−0.22** **−0.35 to −0.10**	7.3±0.21	−0.04 −0.30 to 0.21	7.3±0.09	**−0.22** **−0.31 to −0.14**	8.5 7–9	0.23 0.68
4	6	6.9±0.20	−0.08 −0.31 to 0.16	7.9±0.30	−0.23 −0.45 to 0.01	7.8±0.37	**−0.47** **−0.76 to −0.17**	7.7±0.23	−0.09 −0.25 to 0.07	9.0±0.00	–	7.5±0.14	−0.17 −0.29 to −0.06	7.5 7–8	0.77 0.08
5*	5	6.7±0.29	−0.04 −0.42 to 0.34	7.2±0.31	+0.03 −0.29 to 0.35	7.1±0.28	+0.17 −0.12 to 0.46	7.1±0.24	+0.24 −0.03 to 0.52	5.8±0.36	+0.01 −0.26 to 0.28	7.0±0.14	+0.11 −0.06 to 0.28	7 7–8	0.26 0.77
6	6	7.5±0.24	+0.18 −0.08 to 0.43	7.4±0.33	+0.01 −0.30 to 0.31	8.4±0.23	**+0.28** **0.07 to 0.48**	7.6±0.24	+0.19 −0.01 to 0.39	7.2±0.77	−0.07 −0.32 to 0.19	7.7±0.13	**+0.17** **0.04 to 0.30**	9 8–9	0.18 0.82
7	6	7.5±0.17	+0.07 −0.10 to 0.24	7.2±0.32	−0.01 −0.21 to 0.19	7.4±0.33	**+0.27** **−0.06 to 0.49**	7.7±0.17	+0.01 −0.13 to 0.15	6.8±0.46	−0.04 −0.29 to 0.21	7.5±0.11	+0.08 −0.02 to 0.17	8 8–9	0.00 0.38
8*	2	4.2±0.47	−0.92 −2.59 to 0.76	4.3±0.49	+1.33 0.80 to 1.87	5.4±0.93	+0.46 −0.23 to 1.16	5.4±0.49	0.00 −0.69 to 0.69	8.5±0.29	−0.05 −0.31 to 0.22	4.9±0.29	−0.03 −0.82 to 0.76	5.5 5–6	–
9	4	5.8±0.51	**−0.61** **−1.08 to −0.13**	6.2±0.59	−0.48 −1.15 to 0.19	6.5±0.56	−0.22 −0.69 to 0.25	6.5±0.42	−0.16 −0.48 to 0.16	8.9±0.14	−0.04 −0.31 to 0.22	6.3±0.25	**−0.34** **−0.58 to −0.10**	8 6–9	0.55 0.47
10	6	6.8±0.26	−0.10 −0.39 to 0.18	6.4±0.23	**−0.34** **−0.54 to −0.13**	7.0±0.38	−0.08 −0.35 to 0.19	7.2±0.19	0.00 −0.15 to 0.14	8.8±0.18	−0.05 −0.30 to 0.21	7.0±0.13	−0.08 −0.19 to 0.03	7 6–7	0.63 0.29
11*	4	8.4±0.11	−0.06 −0.24 to 0.12	8.3±0.13	−0.03 −0.25 to 0.18	8.5±0.16	−0.19 −0.41 to 0.04	8.5±0.14	**−0.24** **−0.46 to −0.02**	8.7±0.18	−0.05 −0.32 to 0.21	8.4±0.07	**−0.14** **−0.26 to −0.03**	9 9–9	–
12	5	7.5±0.16	−0.06 −0.21 to 0.08	6.9±0.57	+0.10 −0.18 to 0.38	7.8±0.30	+0.03 −0.16 to 0.23	8.1±0.13	−0.07 −0.19 to 0.04	6.50±0.27	+0.02 −0.28 to 0.24	7.7±0.13	−0.02 −0.11 to 0.06	9 9–9	–
All	61/65 (94%)	7.0±0.09	−0.07 −0.15 to 0.01	7.1±0.12	−0.02 −0.12 to 0.08	7.4±0.11	+0.03 −0.06 to 0.12	7.4±0.07	−0.02 −0.07 to 0.04	7.8±0.15	−0.03 −0.17 to 0.10	7.3±0.04	−0.02 −0.06 to 0.02	8 4–9	0.13 0.24

Bold typeface indicates significance.

*Members who resigned from the Research User Group during the analysis period.

**Figure 2:**
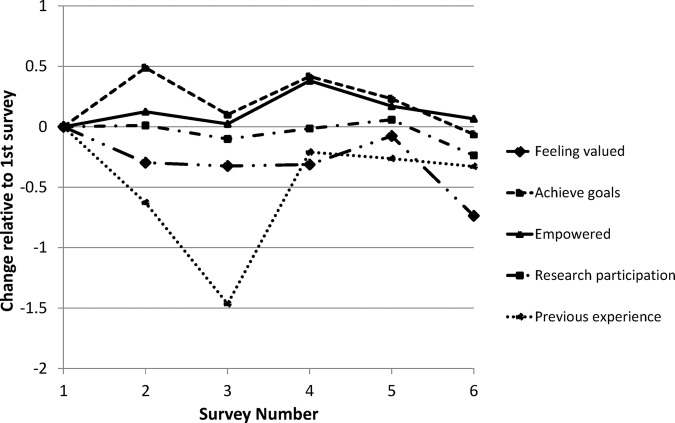
Changes in Likert score over time within factors and over all individuals.

Scores were high for both personal and research factors over the whole group (7.0–7.8, table 2) and factors 2–5 (achieving own goals, empowered, sufficient research support and relevance of previous experience) showed no significant change in score over time (figure 2). However, within factor 1 (being valued) the small decrease in score across the whole group approached significance and occurred mostly between surveys 5 and 6 (−0.07, −0.15 to 0.01, table 2 and figure 2). This was driven by three individuals with a small but significant decline in their scores (1, 3, 9, table 2). One individual reported a significant decrease in their ability to achieve their own goals (10, table 2). Another individual reported a significant decrease in feeling empowered (4, table 2) but two reported a significant increase in empowerment (6, 7, table 2). One individual reported a decline in score for research factors (3, table 2). There was no change in opinion about the value of previous experience over all six surveys, but there was a significant decline in the belief that previous experience was helpful between surveys 1 and 3 (−1.47, −2.58 to −0.35). The RUG followed its own ground rules and this remained stable across all the surveys. Examples of the raw scores and the associated change estimated by the multilevel regression model are shown in table 3 to assist with interpretation.

**Table 3:** Examples of raw scores and resulting change in score estimated by linear regression

Mean change 95% CI	Change in factor 3 (empowered)	Survey number					
		1	2	3	4	5	6
−0.47 (−0.76 to −0.17)	Sig	8,9,9	8,9,9	6,8,9	7,9,9	7,8,9	3,6,8
+0.28 (0.07 to 0.48)	Sig	6,8,9	8,8,9	7,8,9	8,9,9	9,9,9	9,9,9
−0.08 (−0.35 to 0.19)	No change	m,7,9	5,7,9	m,7,8	6,6,8	3,7,8	7,7,8

There was a significant association between responses for personal and research factors (0.41, 0.17 to 0.65). The majority of the variance in the model was explained by the individual and survey number (intracluster correlation coefficient=0.93). Three members of the RUG resigned during the survey period. One individual only completed the questionnaire twice and gave low scores (8, table 2). Another resigning member scored showed no change in score (+0.11, −0.06 to 0.28; 5, table 2) although their expectations score was higher than their mean survey score (5, table 1). A third resigning member showed decreasing satisfaction over time (−0.14, −0.26 to −0.03; 11, table 2). The other two individuals showing a pattern of significantly decreasing scores resigned within 3 months of the survey period (3, 9, table 2). All cited practical reasons for their resignation such as relocation or other time-consuming commitments.

Repeating the analysis using an ordered logistic regression model did not alter the interpretation of the results.

## Discussion

The two main aims of this survey were to apply the questions and theoretical framework recommended by Morrow *et al*^6^ and to evaluate the PPI in Greater Manchester PSTRC from the participant's perspective. Cronbach's α showed acceptable to good internal consistency suggesting that the same underlying concept was addressed by all the questions within the factors (research question 1). With respect to the second research question, the RUG had high expectations of the PPI which were largely met and overall scores representing levels of feeling valued, achieving one's own goals and feeling empowered were high, as were the overall scores representing the quality of the relationships with the researchers and opportunities to participate (third research question). The statistical method allowed viewing of the change in score over time adjusted for the differences in individual absolute scores (research question 4). The modest decline in feeling valued (factor 1) over time needs to be addressed. The high scores created a ceiling effect, thereby reducing the potential to measure increasing scores. For example, in table 3, it is clear that one individual could not record increased empowerment as they were already giving the maximum score of 9, 9, 9. This is always a potential problem when using a finite scale aiming to simultaneously measure absolute and change in score. In future versions of the questionnaire, alternative versions of the scale labels could be tried^9^ or the Likert scale widened or replaced with a visual analogue scale. However, maintaining high scores could be considered positive given that enthusiasm for most activities will naturally wane over time. It is arguable whether or not high expectations should have been encouraged at recruitment as this might lead to unrealistic expectations of the influence of the group.

The association between scores for personal and research factors (research question 5) is consistent with the hypothesis that higher levels of participation in research will lead to higher personal satisfaction but does not provide evidence for a causal relationship. It could equally be argued that individuals with higher levels of personal satisfaction are more likely to look for, or be open to, research opportunities.

The evaluation was intended to highlight any problems at an early stage, so that appropriate action could be taken. A preliminary analysis after the third survey suggested that some participants desired more research opportunities. As a consequence, more involvement opportunities were offered at theme/project level. This coincided with a small overall increase in scores between surveys 3 and 4. However, it is possible that this led to members feeling overburdened leading to a small decrease in scores between surveys 4 and 5.

An important question is whether or not a decrease in scores was observed leading up to the resignation of some RUG members. With hindsight, there were some indicators of dissatisfaction leading up to resignations that occurred during the survey period; one individual failed to meet their expectations score, another had low scores right from the start and one showed an overall decrease in score. The other two individuals showing a pattern of decreasing scores resigned within 3 months of the survey period. RUG members showing similar patterns should receive extra support in future. However, while the questionnaire may be able to retrospectively identify changes in scores, it is less suited to an alert function; at least 3–6 months of data are required to identify a significant change in score. The wide variation of the perceived value of previous experience was surprising; this might be expected to be stable over time (figure 2).

In the discussions with the RUG following distribution of this paper, some valuable insights were gained. One point was that the high response rate might be explained by a sense of obligation due to payment of expenses and it should not be assumed to mean that the questionnaire was acceptable. Another point raised was that they were not involved in the study design, so the questionnaire may not reflect what they believe to be important. One aim of the questionnaire was to provide an objective evaluation based on generalisable concepts (the theoretical framework) rather than the opinions of this specific PPI group, but also there was a practical reason in that the expectations questionnaire had to be designed before the first RUG meeting. Another constructive suggestion was that a question be added addressing whether the level of involvement is burdensome, too little or about right.

Although previous work has defined the norms and values underlying PPI in research,^10–12^ we are not aware of any other quantitative evaluations of the quality of PPI from the perspective of the participants over time. Our approach focuses on norms rather than values such as transparency or moral and ethical concerns. The CIROP tool measures the impact of research partnerships on the community,^13^ whereas we seek to evaluate the quality of involvement in the research process.

This analysis focuses on quality from the perspective of individuals participating in PPI, but analysis is underway to set it within the context of quality in terms of impact and the researcher's perspective. Further work to explore whether the RUG had the factors identified in the theoretical framework in mind when completing the questionnaire is required to provide evidence of face validity for the questionnaire, as well as repeating the Cronbach's α measurements in another PPI group. Future work should address the ceiling effect^14^ and other modifications that will make the questionnaire more responsive, so that it can identify individuals who may benefit from extra support in a more timely fashion.
